# Effect of Titanium and Zirconium Oxide Microparticles on Pro-Inflammatory Response in Human Macrophages under Induced Sterile Inflammation: An In Vitro Study

**DOI:** 10.3390/ma14154166

**Published:** 2021-07-27

**Authors:** Liza L. Ramenzoni, Laura B. Flückiger, Thomas Attin, Patrick R. Schmidlin

**Affiliations:** 1 Clinic of Conservative and Preventive Dentistry, Center of Dental Medicine, University of Zurich, 8032 Zurich, Switzerland; Dr.L.B.Flueckiger@hotmail.com (L.B.F.); Thomas.Attin@zzm.uzh.ch (T.A.); Patrick.Schmidlin@zzm.uzh.ch (P.R.S.); 2 Center of Dental Medicine, Laboratory of Applied Periodontal and Peri-Implantitis Sciences, Clinic of Conservative and Preventive Dentistry, University of Zurich, 8032 Zurich, Switzerland

**Keywords:** inflammation, cytokines, macrophage, peri-implantitis, titanium, zirconia

## Abstract

The wear-debris particles released by shearing forces during dental implant insertion may contribute to inflammatory reactions or osteolysis associated with peri-implantitis by stimulating inflammasome-activation. The study aim was to examine cytotoxic and pro-inflammatory effects of titanium (TiO_2_) and zirconia (ZrO_2_) particles in macrophages regarding their nature/particle concentration over time under sterile lipopolysaccharide (LPS) inflammation. Macrophages were exposed to TiO_2_ and ZrO_2_ particles (≤5 µm) in cell culture. Dental glass was used as inert control and LPS (1 μg/mL) was used to promote sterile inflammation. Cytotoxicity was determined using MTT assays and cytokine expression of *TNF-α*, *IL-1β* and *IL-6* was evaluated by qRT-PCR. Data were analyzed using Student’s *t*-test and ANOVA (*p* ≤ 0.05). Cytotoxicity was significantly increased when exposed to higher concentrations of glass, TiO_2_ and ZrO_2_ (≥10^7^ particles/mL) compared to controls (*p* ≤ 0.05). Macrophages challenged with TiO_2_ particles expressed up to ≈3.5-fold higher upregulation than ZrO_2_ from 12 to 48 h. However, when exposed to LPS, TiO_2_ and ZrO_2_ particle-induced pro-inflammatory gene expression was further enhanced (*p* ≤ 0.05). Our data suggest that ZrO_2_ particles produce less toxicity/inflammatory cytokine production than TiO_2_. The present study shows that the biological reactivity of TiO_2_ and ZrO_2_ depends on the type and concentration of particles in a time-dependent manner.

## 1. Introduction

Dental implants have been a breakthrough healthcare solution since their invention by Brånemark in the 1960s, and are currently a worldwide standard treatment for partial or total tooth loss [1,2,3]. Dental implant use on edentulism treatment routinely presents a success rate of more than 90% [4,5,6]. Nonetheless, peri-implantitis prevalence has been rising on account of mucosa biofilm-related inflammation and alveolar bone dismantling [7,8]. Following previous studies, peri-implantitis prevalence rates typically range from 11% to 53% for patients and from 5% to 37% for implants [9,10,11,12,13]. Strong associations have been found between implant surface oral biofilm microbiota and the host immune response, which leads to the inflammatory pathogenesis of peri-implantitis [14,15]. In fact, immunopathological events that regulate peri-implantitis development were shown to closely mirror those of periodontitis, and even oral bacterial species were found to have strong similarities to periodontitis [16,17]. In addition, peri-implantitis or mucositis may occur on account of previously well-established risk factors, such as smoking, non-controlled type 2 diabetes mellitus, lack of oral hygiene or maintenance, history of periodontitis and obesity [18,19]. These indicators suggest that systemic metabolic disorders should not be neglected when placing a dental implant since they can potentially contribute to peri-implantitis development [20,21]. Titanium-based alloys are considered the gold standard material due to its remarkable mechanical (i.e., oxidation/corrosion resistance) and biological (bone biocompatibility) properties [22,23]. Within the four commercially available pure titanium grades (1 to 4), Ti-6Al-4V is the most widely used for dental implants with the highest oxygen content and best overall mechanical strength. For improving corrosion resistance and reducing elasticity, the zirconium alloy element was added to titanium, creating a stabilizing effect in the β structure (metastable or stable) [22,23]. Despite its biocompatible advantages, questions about titanium sensitivity have been notably arising and it is detected with an estimated prevalence of 0.6% [24]. Previous studies indicated clinical titanium hypersensitivity [24,25]. Animal studies showed accumulation of titanium particles in lymph nodes, lungs and bones after implant placement [26]. In addition, titanium in contact with fluoride or metal alloys in the saliva demonstrated corrosion [27]. However, the clinical relevance of these observations remains unclear. As an alternative to titanium alloy implants, the zirconium dioxide ceramics have been introduced with esthetic improved properties. The currently used tetragonal zirconia (yttrium oxide (yttria)-stabilized zirconia) is the ceramic of choice for dental implants [22,23,28]. The white, opaque color of zirconia, along with good established biocompatibility and low affinity to bacterial plaque, make it a material of interest in dental implantology. Zirconia alloys also present significant physical and mechanical properties as an advanced dental implant material, such as fracture resistance, high flexural strength and corrosion resistance [29]. However, zirconium was shown to slowly develop roughness with time by possible transformation of the tetragonal phase into monoclinic phase, thus inducing progressive material deterioration [29]. Aging is another critical process that can result in microcracking and material stress and can be influenced by macroscopic shape and the surface characteristics during implant production [29]. The structure of the titanium and zirconium alloys’ surface may substantially affect the implant biocompatibility, and in the long-term prospect, they could also have potential toxic effects and cause allergic reactions. Still requiring further particular consideration is the fact that an immunological response may partially also in part occur due to metal or foreign-body particles released by abrasion from shearing forces during dental implant positioning, abutment–implant interface micromovements and biocorrosion [30,31,32,33,34]. Both titanium and zirconium particle elements were histologically present in peri-implantitis mucosa [35,36]. A higher level of metal-like particles was also detected in patients with peri-implantitis lesions [35,36]. Thus, implant wear material particles may indeed play a key role in the implant hypersensitivity or allergic reactions, yet remain currently little understood. The lack of research in this area is not accidental. The design requirements for such a systematic in vitro or in vivo examination are stringent and generally difficult to meet. Many histological studies have demonstrated that peri-implantitis human biopsy material presented metal-like debris or particles and associated inflammatory tissue cell infiltrate around the implant [21,30,37]. However, the same detectable metal particles were also observed at healthy implant sites, which makes it unclear whether the peri-implantitis pathogenesis is associated with the type of particles originating from titanium or zirconia ceramic [35]. Even so, several in vitro studies for the most part confirmed evidence that micro- or nano-particles of common titanium implant alloys may induce cell cytotoxicity and pro-inflammatory augmentation [38,39,40,41]. There is also limited data showing yttria-stabilized tetragonal zirconia particles as one of the implant-released materials that may cause similar inflammatory or cytotoxic effects [42]. This fundamentally disputes the potential function of zirconia particles in the establishment of peri-implantitis. Many cytokines, such as *IL1-β*, *IL-6* and *TNFα*, play a crucial role in the innate immune system after macrophage toll-like receptors are activated by bacterial pathogens [43]. It has been shown that various crystal particles (silica, cholesterol, aluminum, asbestos) can mediate activation and release of *IL1-β* in macrophages [44,45,46,47]. Moreover, endotoxin bacterial products or lipopolysaccharides (LPS) from pathogenic periodontal bacteria, such as *Aggregatibacter actinomycetemcomitans* or *Porphyromonas gingivalis*, are known to increase the expression of cytokines through activation of the inflammasome complex, and consequently boost osteoclastic activity, loosening implants [41]. Most studies use in vitro sterile inflammation by Gram-negative endotoxin LPS, probably because it is very common and easily obtainable, and the most relevant source for peri-implantitis study is *Porphyromonas gingivalis*. As dental implants are progressively considered go-to treatments for tooth loss, the use of LPS sterile inflammation is paramount in order to mimic a peri-implantitis environment for in vitro evaluation of which types or concentrations of implant particles could escalate the inflammatory process, affecting implant tissue surroundings over time.

Therefore, the primary focus of this in vitro study is on assessing the effect of titanium (TiO_2_) and zirconia (ZrO_2_) implant microparticles on the macrophage cytotoxicity and inflammatory stimulating cytokine expression over time. A dental glass (ionomer filling material) was also included in the experimental evaluation as a control, as it is an inert material known for its low (inert) chemical or biological reactivity. In addition, TiO_2_ and ZrO_2_ were also simultaneously employed in a sterile inflammation with endotoxin *Porphyromonas gingivalis* LPS in order to assess the possible surge expression of inflammatory cytokines. We show that cell toxicity enhancement may be caused by increased TiO_2_ and ZrO_2_ concentrations in the cell growth medium, and the LPS may have a synergistic effect on the pro-inflammatory reaction over time.

## 2. Materials and Methods

### 2.1. Cell Culture

Human immortalized monocyte-like leukemia THP-1 cells (ATCC^®^ TIB-202™; American Type Culture Collection, Manassas, VA, USA) were placed in a 6-well plate (5 × 10^5^ cells/well) with RPMI-1640 medium (Roswell Park Memorial Institute, Sigma-Aldrich, Schaffhausen, Switzerland), supplemented with 10% fetal bovine serum and 1% penicillin-streptomycin antibiotics (Invitrogen, Carlsbad, CA, USA) in an incubator with 5% CO_2_/95% air at 37 °C. The THP-1 cells were differentiated to macrophages using phorbol 12-myristate 13-acetate (PMA, Sigma Aldrich, St. Louis, MO, USA), followed by 24 h incubation in RPMI medium. 

### 2.2. Titanium, Zirconium and Glass Microparticles

Commercially pure particles of titanium (IV) dioxide microparticles (TiO_2_, CAS 13463-67-7, <5 μm particle size, Sigma Aldrich, St. Louis, MO, USA) and zirconium (IV) dioxide microparticles (ZrO_2_, CAS 131423-4, <5 μm size, Sigma Aldrich, St. Louis, MO, USA) were used in this study. Inert glass ionomer filling material particles (55 wt% SiO_2_, 10 wt% Al_2_O_3_, 10 wt% B_2_O_3_ and 25 wt% BaO, Schott AG, Mainz, Germany, ident. no: 642905106, lot: M 92605, mean particle size of D50 ≈ 5 μm) were also used as controls [48,49]. Glass particles were kindly donated by Dr. D. Mohn from the Department of Chemistry and Applied Biosciences, Institute for Chemical and Bioengineering, ETH, University of Zurich. Particles of TiO_2_, ZrO_2_ and glass were suspended in ultrapure water and filtered with Millipore filters (Millipore Sigma, Burlington, MA, USA) to a final concentration of 1 mg/mL (stock suspension) in order to obtain particles in a phagocytable range (2–5 μm). The suspensions were prepared by 10 min sonication in an ultrasonic water bath (Sonores Super RK 156 BH, Berlin, Germany) and vortexed for 30 s at full speed. The particles were cleaned using 25% nitric acid for 2 h, followed by 3 washes in 1× phosphate-buffered saline (Invitrogen, Waltham, MA, USA) for 5 min each and placed in 95% ethanol with 0.1 N of NaOH for 24 h, followed by 3 washes in 1× phosphate buffered saline (PBS). Cells were exposed to different particle concentrations in cell cultivation medium, but the same particle number concentrations of TiO_2_ and ZrO_2_ particles was added to the cultured cells. The calculation of the relation between particle mass and particle number concentration was performed and described elsewhere [50]. The ratio between particle number concentration to particle mass concentration was estimated, and the final particle mass concentration was different for the two particle types. Following calculations, the particle number concentrations of 1 × 10^7^, 1 × 10^8^ and 1 × 10^9^ particles/mL represented particle mass concentrations of 0.876, 8.58 and 89.9 μg/mL for TiO_2_ particles and 0.912, 9.11 and 92.6 μg/mL for ZrO_2_ particles, respectively.

### 2.3. Macrophage Differentiation and Particle/LPS Stimulation

For experiments, cells were seeded in 24-well plates at a density of 1 × 10^5^ cells/cm^2^ in a final volume of 1 mL. To differentiate monocytes toward macrophage phenotype, cells were cultivated for 3 days in complete media supplemented with 100 nM phorbol 12-myristate 13-acetate (PMA, Sigma-Aldrich, St. Louis, MO, USA), followed by 24 h cultivation under PMA-free conditions. For the cell viability, THP-1 cells were treated with particle number concentrations of 1 × 10^7^, 1 × 10^8^ and 1 × 10^9^, representing mass concentrations of 0.876, 8.58 and 89.9 μg/mL for TiO_2_ and 0.912, 9.11 and 92.6 μg/mL for ZrO_2_ particles in complete culture medium for 12, 24 and 48 h. Therefore, for the cell viability, the particles were analyzed at concentrations between 0 and 100 μg/mL according to the corresponding particle mass concentration applied during experimentation. Concentration-dependent analysis revealed that 1 × 10^8^ and 1 × 10^9^ particles/mL confirmed adverse toxicity effects on macrophages’ viability. Consequently, the particle number concentration of 1 × 10^7^ particles/mL was finally sublethal, and for this reason, was selected for the gene expression assays. In order to assess particle-induced pro-inflammatory gene expression in THP-1 after 12, 24 and 48 h of stimulation by the different particles TiO_2_, ZrO_2_ and glass (at particle number concentration of 10^7^ particles/mL), cell culture lysates were collected, immediately centrifuged, transferred to a fresh tube and stored at −80 °C until used for RT-PCR assays. Lipopolysaccharides from *Porphyromonas gingivalis* (*P. gingivalis* LPS) were purchased from Sigma-Aldrich. Cells were cultured at 37 °C under 5% CO_2_ with 1 μg/mL of *P. gingivalis* LPS for 24 h. The growth medium was then replaced with 100 μL of RPMI 1640 medium containing the TiO_2_, ZrO_2_ and glass particles at the 10^7^ particles/mL concentrations. Cells were exposed to these agents for a further 48 h to finalize the experiment. Macrophages cultured under particle-free/LPS conditions were used as negative controls.

### 2.4. Cell Viability Assay

To assess the potential influence of TiO_2_, ZrO_2_ and glass microparticles on in vitro cytotoxicity, we performed a tetrazolium (MTT; (3-(4,5-dimethylthiazol-2-yl)-2,5-diphenyltetrazolium bromide, Sigma–Aldrich) dye reduction assay (5 mg/mL in 1× PBS) according to the manufacturer’s instructions. Briefly, monocytes were seeded in 96-well plates at a density of 10^5^ cells/cm^2^ in a final volume of 200 μL and differentiated to macrophages, as described above. Cells were treated for 12, 24 and 48 h with concentrations of 10^7^, 10^8^ and 10^9^ particles/mL of TiO_2_, ZrO_2_ and glass. Negative control cells were cultured under particle-free conditions. After particle stimulation, MTT reagent was added to the cell culture for 1 h, and the concentration of the soluble reduced formazan product was recorded by a light absorbance spectrophotometer reader at 490 nm, and reference absorbance at 630 nm (Biotek Instruments Elx 800, Witec AG, Sursee, Switzerland). 

### 2.5. Gene Expression Analysis

Gene expression analysis was performed by real-time polymerase chain reaction (qRT-PCR) to assess expression levels of defined sets of genes after particle stimulation of the cells. Total RNA was extracted from the cells after particle (10^7^ particles/mL of TiO_2_, ZrO_2_ and glass) and *P. gingivalis* LPS (1 μg/mL) stimulation by using the RNeasy MicroKit (Qiagen, Hilden, Germany). The quality and quantity of the isolated RNA was analyzed using a NanoDrop ND1000 spectrophotometer (Thermo Scientific, Basel, Switzerland). Complementary DNA (cDNA) was synthesized in a volume of 20 μL from 300 ng of total RNA using the iScript cDNA Synthesis Kit (Bio-Rad, Cressier, Switzerland). qRT-PCR reaction was performed using TaqMan’s One-Step Master Mix kit (Applied Biosystems) and specific primers (purchased from Microsynth, Switzerland) for *TNF-α* (forward: 5′-CCG TCT CCT ACC AGA CCA AG-3′, reverse: 5′-CTG AGT CGG TCA CCC TTC TC-3′), *IL-1β* (forward: 5′-ACA GAT GAA GTG CTC CTT CCA-3′, reverse: 5′-GTC GGA GAT TCG TAG CTG GAT-3′) and *IL-6* (forward: 5′-GGT ACA TCC TCG ACG GCA TCT-3′, reverse: 5′-GTG CCT CTT TGC TGC TTT CAC-3′). The comparative Ct method (2^−ΔΔCT^ formula) was used to calculate gene expression levels relative to the housekeeping gene *GAPDH* (forward: 5′-GCT CTC TGC TCC TCC CTG TT-3′, reverse: 5′-CAC ACC GAC CTT CAC CAT CT-3′) and normalized to control cells (with no particles). All samples were tested in triplicate, and 3 independent experiments were performed. The results were presented as means ± standard deviations.

### 2.6. Statistical Analysis

The mean values and standard deviations were computed for the MTT test, and a multiple comparison analysis of variance (ANOVA) with Bonferroni adjustment with a global significance level of 5% was conducted to assess the statistical significance of the differences between the experimental groups using IBM SPSS software (IBM SPSS Statistics for Windows, version 23.0; IBM Corp., Armonk, NY, USA). Differences were considered significant at *p* ≤ 0.05, and all experiments were performed in triplicate and repeated at least three times under the same conditions.

## 3. Results

### 3.1. Effect of Particles on Cell Viability

**Figure 1:**
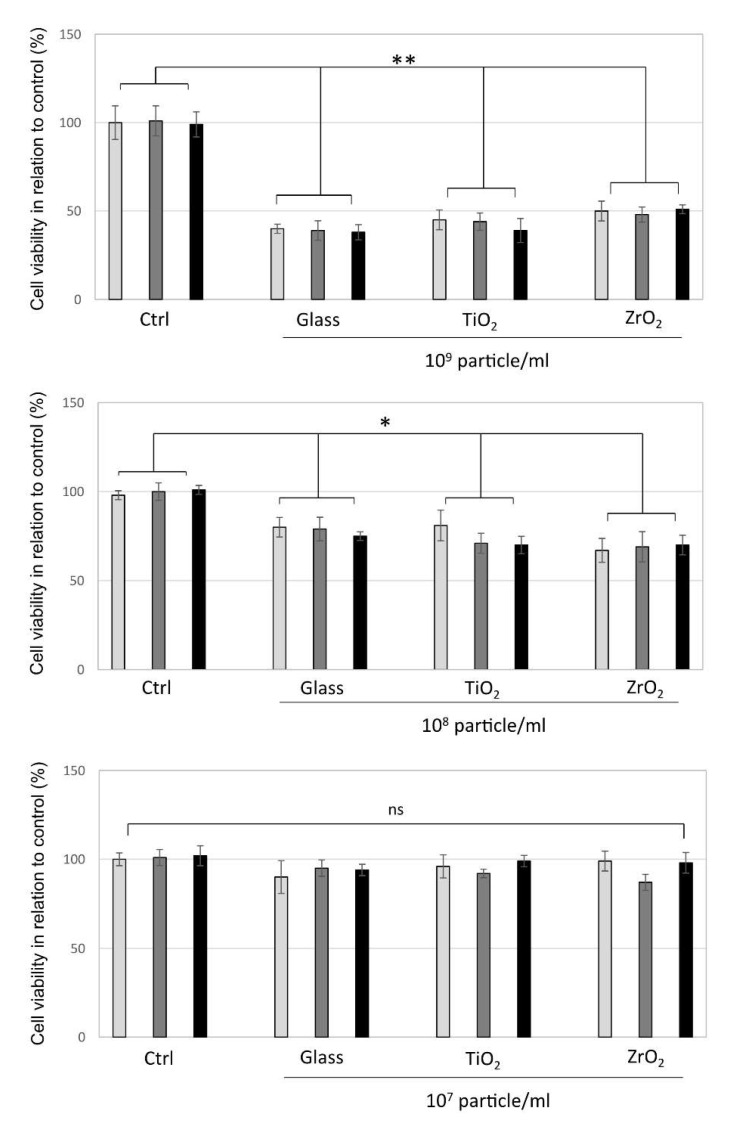
Evaluation of cellular viability (MTT assay). Cytotoxicity of human macrophages (THP-1) over 12, 24 and 48 h for the untreated control (Ctrl), glass, titanium (TiO_2_) and zirconia (ZrO_2_) particles at 10^9^, 10^8^ and 10^7^ particles/mL concentrations in medium. Results are presented as percent of control (mean ± SD) from three independent experiments. * *p* ≤ 0.05, ** *p* ≤ 0.001, ns: No statistical differences were detected using one-way ANOVA.

To investigate the influence of different concentrations of TiO_2_, ZrO_2_ and inert glass microparticles on cell viability, we determined the cytotoxicity of these particles in various particle concentrations of 10^7^, 10^8^ and 10^9^ particles/mL using the MTT assay on the cultures of phorbol 12-myristate 13-acetate (PMA)-differentiated THP-1 cells. After 12, 24 and 48 h of exposure, macrophage viability was significantly reduced when exposed to higher concentrations of TiO_2_ and ZrO_2_ particles (≥10^7^) compared to the untreated negative controls and glass (*p* < 0.05). However, at concentration ≤ 10^7^, all three particles showed no cytotoxicity effect in this cell culture assay (Figure 1). Increased cytotoxicity of glass at high concentrations ≥ 10^7^ particles/mL is similar to TiO_2_ and ZrO_2_ at 12, 24 and 48 h; however, all particles are less cytotoxic at concentrations ≤ 10^7^ after 48 h of application. The particle concentration of 10^7^ was selected for further gene expression analysis since enhanced gene expression was detected in the samples with reduced cytotoxicity or sub-toxic conditions.

### 3.2. Particle-Induced Pro-Inflammatory Gene Expression in Macrophage Cultures Over Time

**Figure 2:**
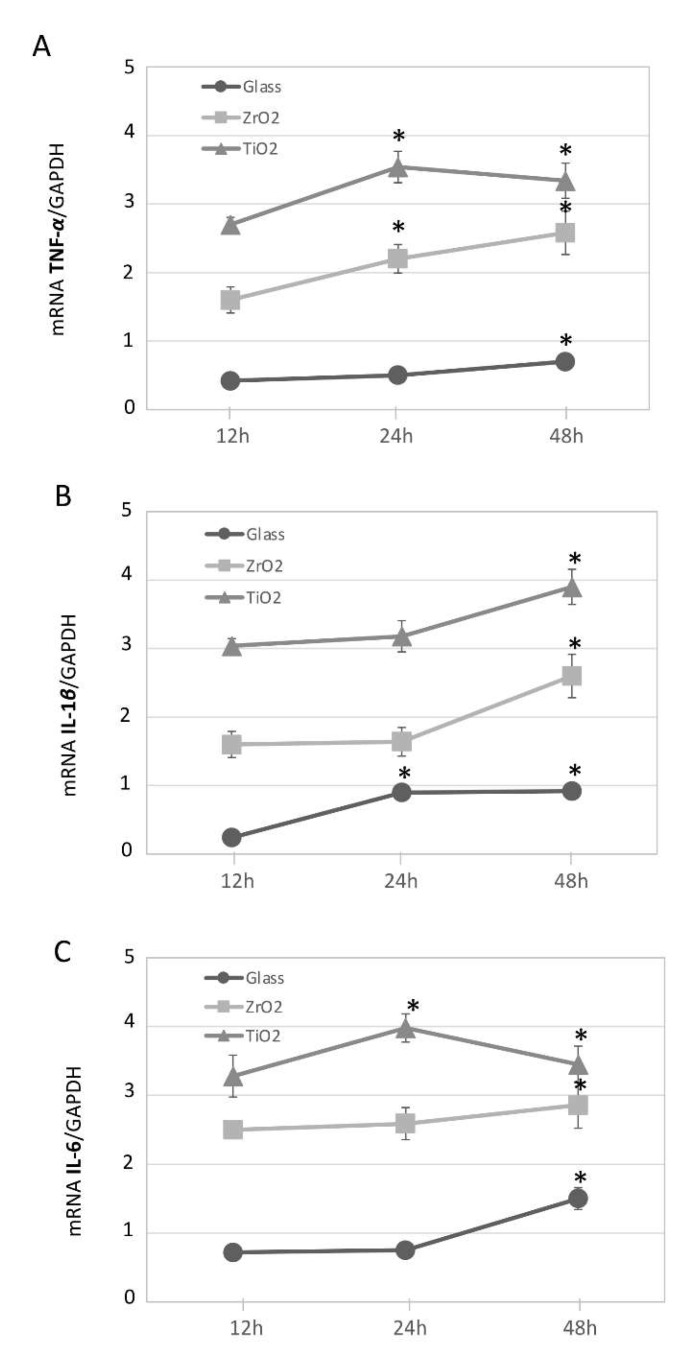
Particle-induced pro-inflammatory gene expression in THP-1 over time. Expression of mRNA for inflammatory cytokines in THP-1 macrophages challenged with titanium, TiO_2_ (▲), zirconia, ZrO_2_ (◼), and glass (●) particles (10^7^ particles/mL concentration). qRT-PCR, with normalization to GAPDH using the Ct method analysis, are shown as means ± SD. (**A**) *TNF-α*, (**B**) *IL-1β*, (**C**) *IL-6*. The symbol * indicates a statistically significant increase in cytokines’ mRNA expression in comparison to non-challenged macrophages. There is a statistically significant interaction between time and material in all cases (two-way repeated measure ANOVA). * *p* ≤ 0.05.

To obtain a cytokine expression profile as a function of TiO_2_, ZrO_2_ and glass particles on differentiated macrophages, we performed a gene expression analysis of inflammatory molecules over a period of time. Differentiated macrophages exposed to the 10^7^ particles/mL concentration for 12, 24 and 48 h were analyzed toward pro-inflammatory phenotype. Relative expression levels of selected marker gene sets consisting of cytokines (*TNF-α*, *IL-1β* and *IL-6*) were assessed. Results were set in relation to differentiated macrophages cultured under particle-free conditions as negative controls. Cultured macrophages challenged with TiO_2_ and ZrO_2_ particles expressed an increase in mRNA for inflammatory cytokines *TNF-α*, *IL-1β* and *IL-6* (up to ≈3.5-fold upregulated) at 12, 24 and 48 h (Figure 2A–C, as indicated by the symbol *). However, compared to TiO_2_, ZrO_2_ particles and glass produced a significantly lower increase of pro-inflammatory gene expression (*p* < 0.05), and glass particles presented an even lower effect compared to TiO_2_ and ZrO_2_. There was a statistically significant interaction between material and time (*p* < 0.05).

### 3.3. Particle and Sterile LPS Inflammation-Induced Pro-Inflammatory Gene Expression

**Figure 3:**
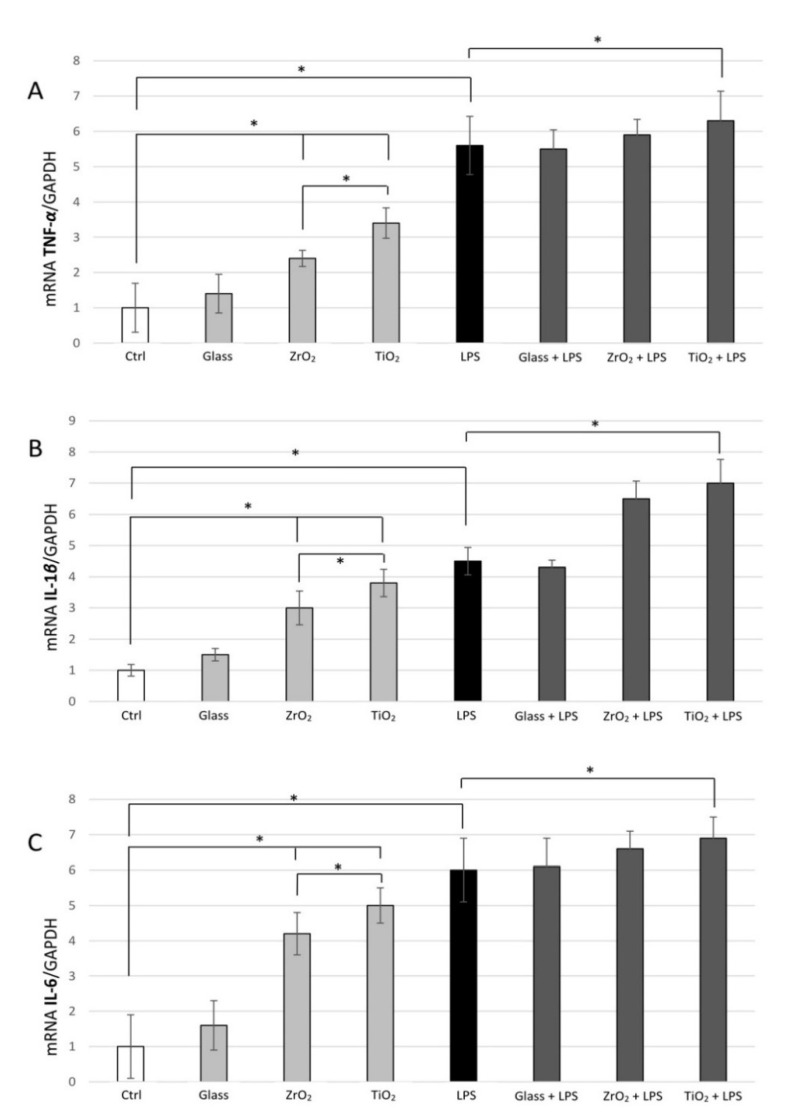
Particle and sterile LPS inflammation-induced pro-inflammatory gene expression in THP-1. Expression of mRNA for inflammatory cytokines in macrophages challenged with titanium (TiO_2_), zirconia (ZrO_2_) and glass particles under sterile inflammation (LPS = 1 μg/mL) in 48 h. qRT-PCR, with normalization to GAPDH using the Ct method analysis, are shown as means ± SD. (**A**) *TNF-α*, (**B**) *IL-1β*, (**C**) *IL-6*. The symbol * indicates a statistically significant increase in cytokines’ mRNA expression in comparison to non-challenged macrophages. * *p* ≤ 0.05.

Particles were used at a non-toxic 10^7^ particles/mL concentration. Cytokines’ expression was more enhanced under exposure to TiO_2_ in comparison to the control (*p* < 0.001). No significance was found between control and glass (*p* > 0.05), and the exposure to TiO_2_ caused higher upregulation than to ZrO_2_ (*p* < 0.001). This effect was dependent on the type of particle as, for instance, TiO_2_ presented higher expression of inflammatory genes compared to ZrO_2_ or glass particles (*p* < 0.05). Adding *P. gingivalis* LPS (1 μg/mL) to the THP-1 cells for 24 h (before exposure to TiO_2_, ZrO_2_ and glass particles) caused an up to 6-fold increase in *TNF-α* (Figure 3A), *IL-1β* (Figure 3B) and *IL-6* expression (Figure 3C) in comparison to the control (*p* < 0.001). After an additional 24 h exposure to *P. gingivalis* LPS, a statistically significant higher secretion of *TNF-α*, *IL-1β* and *IL-6* was found in cells exposed to particles of TiO_2_ or ZrO_2_ in comparison to LPS treatment alone (*p* < 0.001). However, between TiO_2_ and ZrO_2_, no difference was found in this expression with the addition of LPS (*p* > 0.05).

## 4. Discussion

The history of implant development shows that throughout the years, much has been improved on implant biomaterial properties and their surfaces. Microscopic implant material wear debris or particles may still be produced by shear forces and abrasion during implant placement, which could steer an inflammatory process, bone loss and subsequent clinical complications [38]. In fact, titanium wear particles have been associated with the establishment of inflammation by stimulating macrophage response [38,39,48], which may be exacerbated by the presence of periodontal bacteria [40,41]. A metal-free implant material alternative to titanium was also proposed for prosthetic rehabilitation, but zirconia wear particles might further affect macrophage stimulation and have not yet been researched in detail. This study attempts to synthetize the effect of different concentrations of TiO_2_ and ZrO_2_ microparticles on human macrophage response. Another purpose of this study was to examine the particles’ combined effect with *P. gingivalis* LPS as a potent endotoxin pathogen for inflammation in peri-implantitis disease.

The findings showed that glass, TiO_2_ and ZrO_2_ particles in higher concentrations presented a detrimental effect on the macrophage cell viability when compared with the respective negative control groups, and that was independent of time exposure. In fact, this concentration-dependent analysis with cell viability evaluation showed that cytotoxicity was not affected at the 10^7^ particles/mL concentration. Therefore, this particle number concentration was also selected as the ideal sublethal concentration for further gene expression assays. As confirmed previously, an increase in the gene expression can be measured when TiO_2_ particles are applied in non-cytotoxic doses [40]. The cell viability assessment presented here is confirmed by previous analyses on cell toxicity of TiO_2_ [38,39,40,41,42,51,52] and ZrO_2_ particles [38]. Similar negative effects were previously shown for human THP-1 cells, which demonstrated higher cytoplasmatic assimilation of nanoparticles within 24 h of exposure [53,54]. As predicted and supported by the literature [55], no reduction in number of viable cells was observed with the tested inert glass when exposed to non-cytotoxic concentrations. The cell viability was further complemented by challenging macrophages with TiO_2_ and ZrO_2_ particles, which finally induced overtime upregulation of pro-inflammatory cytokines *TNF-α*, *IL-1β* and *IL-6*. These cytokines are key molecules in bone remodeling, and enhanced levels of these molecules are commonly found in tissue fluids of degenerative diseases, such as periodontitis and peri-implantitis [43]. In addition, the data showed that stimulation of LPS intensified the cytokine expression even more. However, noticeable quantitative gene expression differences between TiO_2_ and ZrO_2_ was observed, as TiO_2_ particle exposure promoted higher pro-inflammatory gene expression than ZrO_2_ particles in macrophages. This could be explained by the fact that macrophages’ uptake of TiO_2_ particles is more effective than ZrO_2_ particles, which thereby would effectively stimulate the acute activation of an inflammatory gene expression program [51]. The amounts of cytokines were significantly increased for TiO_2_ and ZrO_2_ compared to unstimulated cells. However, the amounts of cytokines from LPS-stimulated cells were slightly lower in the presence of glass compared to the control stimulated with LPS. This result corroborates previous studies by showing the potential antibacterial ability of these glasses to inhibit the secretion of inflammatory cytokines in the presence of an inflammatory stimulus such as LPS Gram-negative bacteria [41,55,56]. The obtained data also confirms earlier research that elucidates the effect of TiO_2_ particles associated with *P. gingivalis* LPS34.

Within the limitations of the present study, we hypothesize that soluble LPS could bind and accumulate on the titanium and zirconia particles from implant insertion debris to induce an inflammatory response, since dissolved LPS and its lipid A-active group may be freely available for macrophage activation in the surrounding implant tissues. It should also be noted, however, that only the response of the THP-1 macrophage cell line was tested here. Future studies are still necessary to compare the response of other cell types of human origin to LPS sterile-induced inflammation not only with titanium or zirconia, but also with other implant or abutment materials. Moreover, nanoparticles and/or micro-particles influence assessment on peri-implant supporting bone destruction is still necessary, especially during early stages of osteointegration. Our present findings support theories for the cause of peri-implantitis which suggest that inflammation is induced not only by microbes, but also by foreign-body reactions to implant material [8,55].

In summary, within constraints, the results provide evidence that titanium and zirconia microparticles may prompt macrophage cytotoxicity by increasing their concentration, and their induced proinflammatory effect can be exacerbated by the presence of LPS in the medium. Additionally, the findings suggest that compared to TiO_2_ particles, ZrO_2_ particles caused reduced toxicity and inflammatory cytokine production. Further studies are necessary to determine whether these responses are likely to be the same in vivo.

## Data Availability

The data presented in this study are available on request from the corresponding author.
